# Aktiia cuffless blood pressure monitor yields equivalent daytime blood pressure measurements compared to a 24-h ambulatory blood pressure monitor: Preliminary results from a prospective single-center study

**DOI:** 10.1038/s41440-023-01258-2

**Published:** 2023-04-03

**Authors:** Tiago P. Almeida, Meritxell Cortés, David Perruchoud, Jérémy Alexandre, Pascale Vermare, Josep Sola, Jay Shah, Luisa Marques, Cyril Pellaton

**Affiliations:** 1 Aktiia SA, Neuchâtel, Switzerland; 2 Division of Cardiology, Mayo Clinic Arizona, Phoenix, AZ USA; 3 Division of Cardiology, Réseau Hospitalier Neuchâtelois (RHNe), Neuchâtel, Switzerland

**Keywords:** Cuffless blood pressure, Hypertension, Ambulatory blood pressure monitor, Optical blood pressure monitor, Continual blood pressure monitoring, Daytime blood pressure

## Abstract

In this preliminary study, we compared daytime blood pressure (BP) measurements performed by a commercially available cuffless—and continual—BP monitor (Aktiia monitor, Neuchâtel, Switzerland) and a traditional ambulatory BP monitor (ABPM; Dyasis 3, Novacor, Paris, France) from 52 patients enrolled in a 12-week cardiac rehabilitation (CR) program (Neuchâtel, Switzerland). Daytime (9am–9pm) systolic (SBP) and diastolic (DBP) BP from 7-day averaged data from Aktiia monitor were compared to 1-day averaged BP data from ABPM. No significant differences were found between the Aktiia monitor and the ABPM for SBP (μ ± σ [95% confidence interval]: 1.6 ± 10.5 [−1.5, 4.6] mmHg, *P* = 0.306; correlation [*R*^2^]: 0.70; ± 10/ ± 15 mmHg agreements: 60%, 84%). Marginally non-significant bias was found for DBP (−2.2 ± 8.0 [−4.5, 0.1] mmHg, *P* = 0.058; *R*^2^: 0.66; ±10/±15 mmHg agreements: 78%, 96%). These intermediate results show that daytime BP measurements using the Aktiia monitor generate data comparable to that of an ABPM monitor.

## Abstract (web summary)

In this preliminary study, daytime blood pressure (BP) measurements performed by a commercially available cuffless – and continual – BP monitor (Aktiia) generated equivalent data compared to a traditional 24-h ambulatory BP monitor (ABPM). The improved general usability of Aktiia over ABPM suggest Aktiia can safely replace ABPM for long-term BP monitoring.

## Graphical abstract

**Figure:**
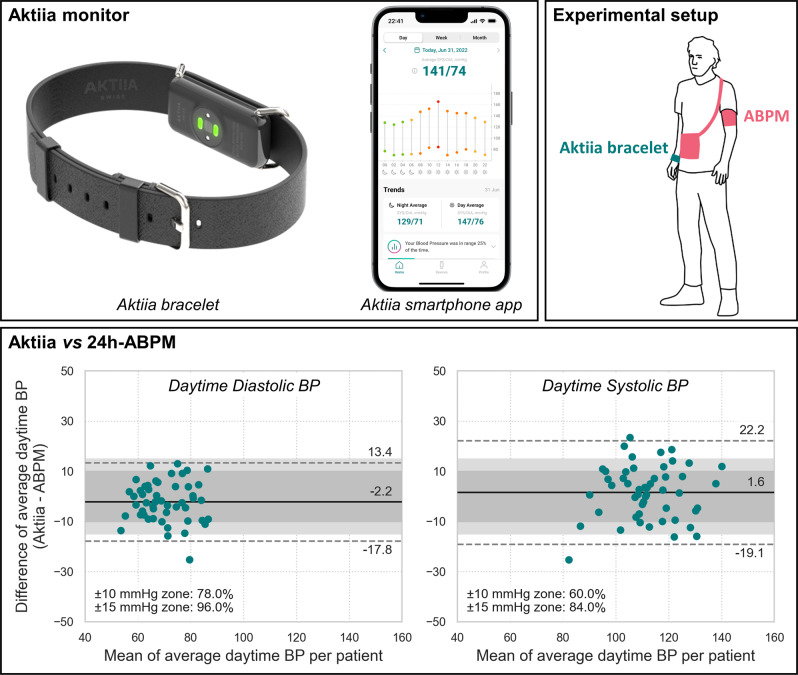
In this preliminary study, daytime blood pressure (BP) measurements performed by a commercially available cuffless – and continual – BP monitor (Aktiia) generated equivalent data compared to a traditional 24-h ambulatory BP monitor (ABPM). The improved general usability of Aktiia over ABPM suggest Aktiia can safely replace ABPM for long-term BP monitoring.

## Introduction

Diagnosis of hypertension and blood pressure (BP) monitoring are broadly based on daytime measurements [1–3]. Traditional modalities for BP measurement use cuff-based oscillometric sphygmomanometers [4]. They are inconvenient, uncomfortable, and episodic by nature [5]. Ambulatory BP monitors (ABPM) are sometimes used to measure systolic BP (SBP), diastolic BP (DBP), and heart rate (HR), but limited to 24–48-hour snapshots [5].

**Fig. 1:**
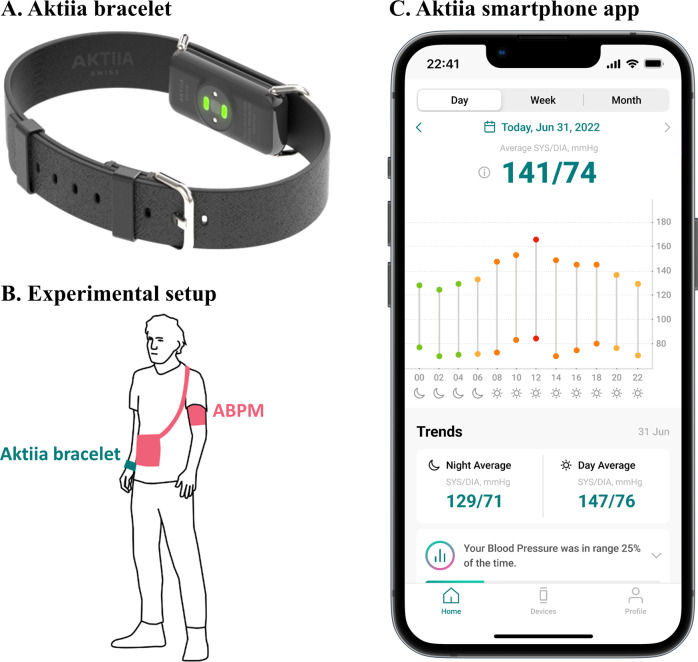
Illustration of Aktiia monitor. **A** Aktiia bracelet with embedded optical sensors. **B** Representation of the experiment setup with Aktiia bracelet on patient’s wrist (green) and the ABPM device (red). **C** Aktiia application displays BP measurements on a smartphone

Some cuffless BP devices overcome the inherent limitations of cuff-based BP monitors, allowing for long-term continual BP monitoring [5]. Aktiia SA (Aktiia, Neuchâtel, Switzerland) has developed a validated CE-marked, non-invasive, cuffless BP monitor that measures BP data following an initialization process using an oscillometric BP device (Fig. 1) [6–8]. Optical sensors embedded in a small bracelet collect photoplethysmography (PPG) signals acquired on the user’s wrist. Following the initialization process, pulse wave analysis is applied to the PPG signals to estimate BP data, which is displayed in a smartphone application. This fully automated device generates no discomfort nor inconvenience for the patient, which makes the Aktiia monitor ideal for long-term continual BP monitoring in daily conditions.

In the present work, we compared daytime BP measurements performed by Aktiia monitor *versus* traditional ABPM. This represents preliminary results from the clinical trial NCT04548986 [9].

## Methods

### Study population

At the time of this analysis, 52 out of 63 estimated total patients (age 53.2 ± 7.1 years, 18.9% female, arm circumference 28.5 ± 2 cm, weight 80.5 ± 13.8 kg, height 173.6 ± 8.1 cm) had been enrolled in a 12-week cardiac rehabilitation (CR) program (Réseau Hospitalier Neuchâtelois, Neuchâtel, Switzerland) following myocardial infarction or heart surgery. The patients used the Aktiia monitor during the 12 weeks of the CR program, while 24-hour ABPM (Dyasis 3, Novacor, Paris, France) was performed twice – on the first and last day of the CR program.

The clinical trial was approved by local ethics committee and all patients provided written informed consent.

### Measurements with the BP modalities

Average daytime (9am–9pm) SBP, DBP, and HR were calculated for 1 day of ABPM and for 7 days of Aktiia monitor, at the beginning and at the end of the CR program. The 7-day average for the Aktiia monitor provides a comprehensive representation of BP for one week in the lives of the patients. For each patient, measurements were organised in two sessions of paired data. The first session consisted of the ABPM collected on the first day of CR compared with the data collected by Aktiia monitor on the first week of CR. Similarly, the second session consisted of the ABPM data collected on the last day of CR compared with the data collected by Aktiia monitor on the last week of CR.

Only sessions that had a minimum of 20 valid daytime ABPM measurements were included in the present study, following recent guidelines [10]. An adapted criterion was implemented for Aktiia monitor to consider patient compliance: only sessions that had a minimum of 20 valid daytime measurements in total during one week, with at least one measurement per day, were considered in the study.

The clinical trials performed with the Aktiia monitor have shown that the calibration is reliable for 1 month and, therefore, it is the recommendation from Aktiia that calibration should be conducted at least once a month [8]. During the first session (the first week of CR), the calibration of the Aktiia monitor was performed on Day 1 for all subjects, *i.e*., on the same day of the ABPM measurements. Multiple calibrations (recalibrations) were performed by the subjects during the 12 weeks of CR after the first calibration performed on Day 1. As a result, the date for recalibration varies for the second session (last week of CR). However, measurements performed by the Aktiia monitor during the second session were always within 30 days of the latest recalibration, as required by Aktiia’s regulatory statements [8].

Further details on the measurement configurations, number of readings, and BP values measured per modality have been included in the Supplementary Materials.

### Statistical Analysis

The results of the comparisons are expressed as mean ± standard deviation (*μ* ± *σ*) and 95% confidence interval (CI) of BP differences, in addition to Student’s paired t-tests.

Population-level differences in BP measurements between Aktiia monitor and ABPM were investigated with scatter plots and Pearson correlation (*R*^*2*^). Similarly, Bland-Altman plots were created to investigate individual-level measurements. Regions of interest (ROI) within ±10 mmHg and ±15 mmHg were included to assess levels of agreement [11].

*P*-values of less than 0.05 were considered statistically significant.

## Results

**Fig. 2:**
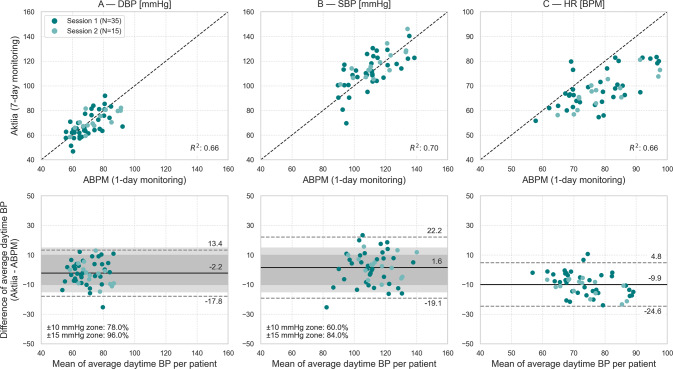
Scatter plots (upper) and Bland-Altman plots (bottom) comparing daytime BP measurements performed by Aktiia monitor (7-day average) and ABPM (1-day average). **A**. Comparison of daytime DBP. **B**. Comparison of daytime SBP. **C**. Comparison of daytime HR. Pearson’s correlation (*R*^*2*^) between the two modalities is shown on the bottom right of the scatter plots. ROI within ±10 mmHg and ±15 mmHg are highlighted in dark grey and light grey, respectively. The percentage of agreement for each region is shown on the bottom left of the Bland-Altman plots. The solid black line denotes the average mean of differences, while the dotted lines denote the limits of agreement (±1.96*σ*). BPM – beats per minute. The dark green dots represent first week sessions (35 sessions), and the light green dots represent last week sessions (15 sessions)

Fig. 2 summarises the results. The original 52 patients resulted in 90 sessions, out of which 50 (56%) had 20 or more valid daytime measurements on ABPM (34 ± 4) and Aktiia monitor (103 ± 61). These sessions were included in the study (details on the exclusion of patients in Supplementary Materials).

Marginally non-significant bias was found between Aktiia monitor and ABPM for daytime DBP (Fig. 2A) (*μ* ± *σ* [95% CI]: −2.2 ± 8.0 [−4.5, 0.1] mmHg, *P* = 0.058; *R*^*2*^ = 0.66). Additionally, the two modalities resulted in agreements of 78 and 96% within the ± 10 mmHg (dark grey) and the ± 15 mmHg (light grey) ROI, respectively. Similarly, no significant differences were found for daytime SBP (Fig. 2B) (1.6 ± 10.5 [−1.5, 4.6] mmHg, *P* = 0.306; *R*^*2*^ = 0.70), with agreements of 60 and 84% within the ± 10/± 15 mmHg ROI, respectively.

HR (Fig. 2C) was significantly lower for Aktiia monitor measurements compared to ABPM (−9.9 ± 7.5 [−12.0, −7.7] bpm, *P* < 0.0001; *R*^*2*^: 0.66).

## Discussion

In the present work, we show preliminary results of a comparison of daytime BP measurements performed by a commercially available cuffless BP monitor and a traditional 24-hour ABPM.

### BP measurements with Aktiia monitor

On a population-level, one week of Aktiia monitoring compared well to 24-h of ABPM monitoring, with no significant bias in the estimation of daytime SBP and DBP averages, converging to agreements within ± 15 mmHg close—or superior—to 85% [11–14].

HR measured by Aktiia monitor was significantly lower compared to ABPM. Aktiia monitor only collects PPG data when the patient’s wrist is still for 30 seconds, while ABPM measures BP data independent of the patient’s level of activity, which might induce misleading BP readings. The results support the perspective that Aktiia monitor measures BP data under more appropriate conditions—*i.e*., while the user is motionless—resulting in lower HR measured by Aktiia monitor.

### Clinical impact

The results suggest that 1-week Aktiia monitor yields equivalent daytime BP values to those obtained via 24-h ABPM, which supports the use of Aktiia monitor for long-term, continual daytime BP monitoring. Assuming patient’s compliance that results in sufficient measurements in one week, Aktiia monitor can safely replace ABPM. The greatly improved general usability of Aktiia over ABPM supports its potential for long-term BP monitoring. Aktiia’s improved comfort and better patient experience likely would improve adherence and adoption. Additionally, clinicians would have easier access to the BP data collected during a one-week period—or even longer—which are critical in diagnosis and monitoring of hypertension.

## Limitations

The present work represents preliminary data from an ongoing prospective clinical study. We believe, however, the results are significant since they represent 84% of the recruited population (52 out of 63 patients). The patients underwent cardiovascular events (myocardial infarction, heart surgery) prior to CR. All patient were treated at the time of inclusion, leading to mostly normotensive patients analyzed in this study.

The current intermediate analysis included only patients that presented 7 days of continual data collection with Aktiia monitor. The implementation of the exact setup in real-life monitoring conditions might require some patients to wear the Aktiia monitor for longer in order to obtain enough measurements.

## Supplementary information

Supplementary Materials
