# Supplementary material for: Aktiia cuffless blood pressure monitor yields equivalent daytime blood pressure measurements compared to a 24-h ambulatory blood pressure monitor: Preliminary results from a prospective single-center study
Source: Hypertens Res. 2023 Apr 3;46(6):1456–61. doi: 10.1038/s41440-023-01258-2 (PMC10239726; doi:10.1038/s41440-023-01258-2)
Supplement: Supplementary file 1 — Supplementary Materials [file 41440_2023_1258_MOESM1_ESM.docx]

**Aktiia Cuffless Blood Pressure Monitor** **Yields Equivalent Daytime Blood Pressure Measurements Compared to a 24-h Ambulatory Blood Pressure Monitor: Preliminary Results from a Prospective Single-Centre Study**

Tiago P Almeida, PhD*; Meritxell Cortés, PhD*; David Perruchoud, PhD*; Jérémy Alexandre, MSc*; Pascale Vermare, MSc*; Josep Sola, PhD*; Jay Shah, MD FACC*‡;

Luisa Marques, BSN§; Cyril Pellaton, MD§

*Aktiia SA, Neuchâtel, Switzerland; ‡Division of Cardiology, Mayo Clinic Arizona, Phoenix, AZ, United States; §Division of Cardiology, Réseau Hospitalier Neuchâtelois (RHNe), Neuchâtel, Switzerland.

**Supplementary Materials**

**Address for correspondence:**

Aktiia SA

Rue du Bassin 8a, 2000 Neuchâtel, Switzerland

Email: publication@aktiia.com

# Methods

## Measurements with the BP modalities

Average daytime (9am-9pm) SBP, DBP and HR were calculated for both 24-h ambulatory BP monitor (ABPM) and 7-day Aktiia monitor at two different instances: the first and last week of a 12-week cardiac rehabilitation (CR) program, as illustrated in Supplementary Figure 1.

The measurements in the two modalities are triggered differently, therefore it is not technically possible to match the exact time of the measurements performed by the BP devices. Consequently, we resorted to daytime averaging of the measurements performed by both modalities, which allowed us to pair both methods. Specifically, the BP measurements performed by both modalities were matched by the week of measurement, and were organized in two sessions of paired data. The first session consisted of the ABPM collected on the first day of CR compared with the data collected by Aktiia monitor on the first week of CR. Similarly, the second session consisted of the ABPM data collected on the last day of CR compared with the data collected by Aktiia monitor on the last week of CR. Accordingly, the daytime average of the 24-h ABPM measurements collected during the first day of the first week of the CR program were matched with the 7-day daytime average of the Aktiia measurements collected during the first week of the CR program. A similar matching was performed for the last day (ABPM) and last week (Aktiia monitor) of the CR program. The averaged BP values from both ABPM and Aktiia monitor were analyzed in pairs, also illustrated in Supplementary Figure 1.

Only sessions that had a minimum of 20 valid daytime ABPM measurements were included in the present study, following recent guidelines [[1](#_ENREF_1)]. An adapted criterion was implemented for Aktiia monitor to consider patient compliance: only sessions that had a minimum of 20 valid daytime measurements in total during one week, with at least one measurement per day, were considered in the study.


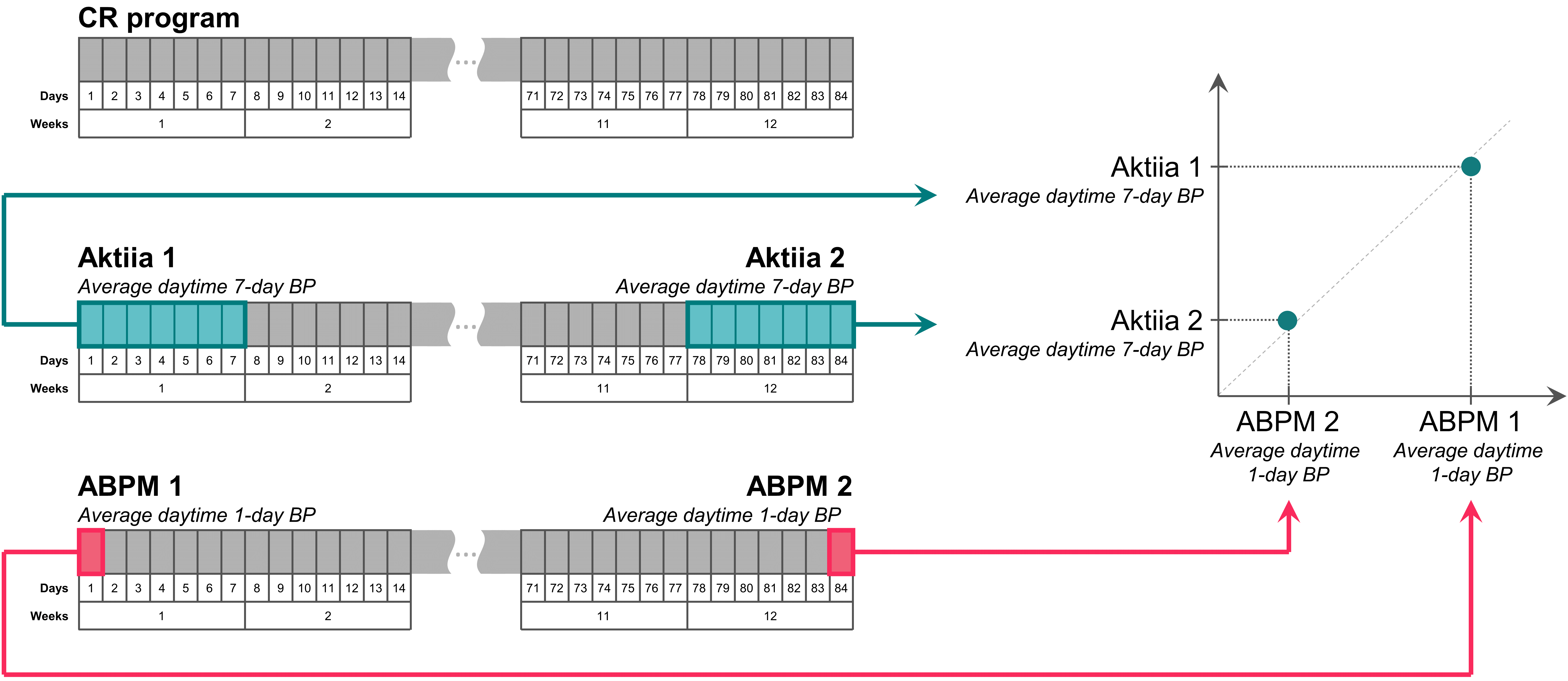


**Supplementary Figure 1.** Illustration of the protocol of BP measurement for ABPM and Aktiia monitor.

The Aktiia monitor performed 14.6 ± 10.8 [7-19] ($\mu\pm\sigma$ [IQR]) daytime readings per day per subject, while ABPM performed 33.7 ± 4.7 [32-37] daytime readings per day per subject (Supplementary Figure 2). In total, the Aktiia monitor performed 102.7 ± 60.8 [64-120] daytime readings during the seven days of monitoring per subject (Supplementary Figure 3). Therefore, the 7-day average for the Aktiia monitor provides a comprehensive representation of BP for one week in the lives of the patients. Figures S2 and S3 illustrate the distributions of quantity of readings performed by the Aktiia monitor and the ABPM. Figures S2 and S3 highlight that only sessions that had a minimum of 20 valid daytime ABPM measurements and only sessions that had a minimum of 20 valid daytime measurements in total during one week, with at least one measurement per day, were included in the present study.

The overall 7-day daytime SBP values measured by the Aktiia monitor were 114.6 ± 13.9 [106-124] mmHg, and the overall 1-day daytime SBP values measured by the ABPM were 112.5 ± 20.5 [99-123] mmHg. Similarly, the overall 7-day daytime DBP values measured by the Aktiia monitor were 70.7 ± 10.5 [63-79] mmHg, and the overall 1-day daytime DBP values measured by the ABPM were 72.5 ± 15.3 [62-83] mmHg. The distribution of BP values per modality are illustrated on Supplementary Figure 4.


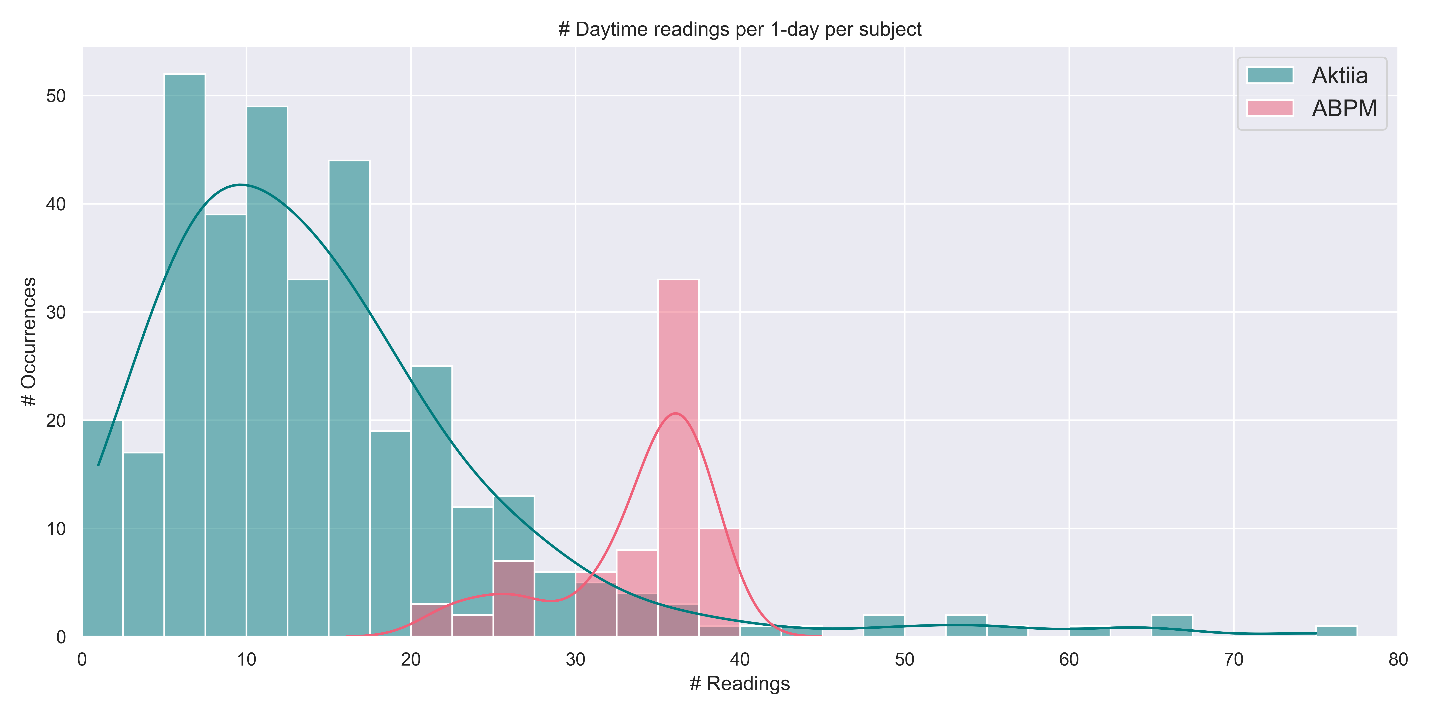


**Supplementary Figure 2.** The amount of readings performed by the Aktiia monitor (green) and ABPM (red) per day per subject.


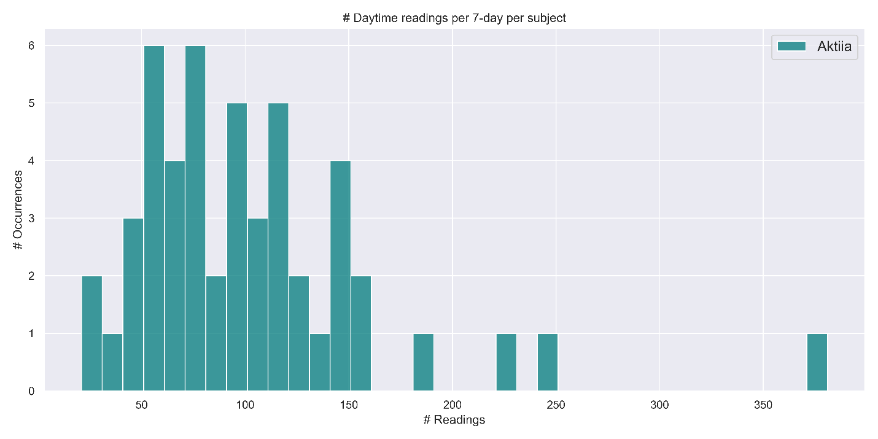


**Supplementary Figure 3.** The amount of readings performed by the Aktiia monitor per 7 days per subject.


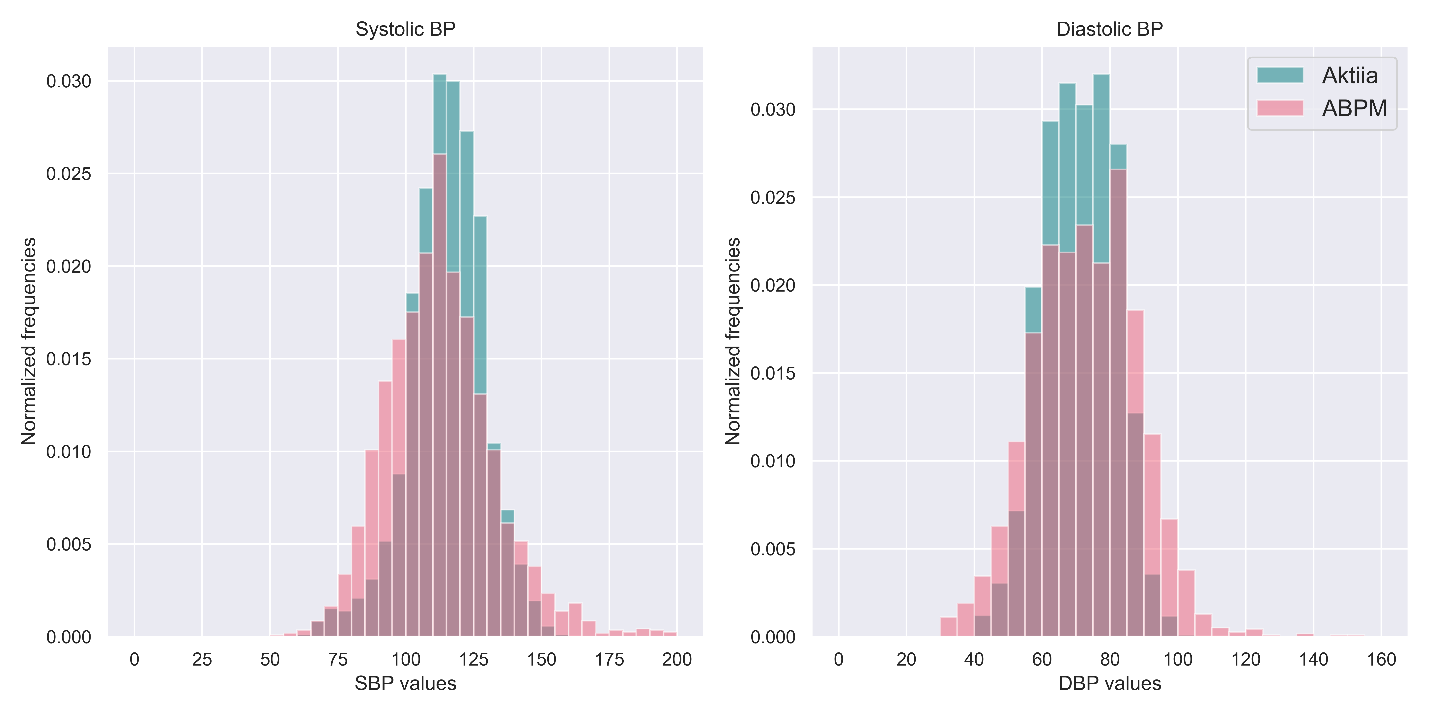


**Supplementary Figure 4. Left panel**: SBP values as measured by the Aktiia monitor (green, 7-day daytime data) and by the ABPM (red, 1-day daytime data). **Right panel**: DBP values as measured by the Aktiia monitor (green, 7-day daytime data) and by the ABPM (red, 1-day daytime data).

The few bins above 50 readings per day (from 4 unique users) in the histogram of Supplementary Figure 2 and the few bins above 200 readings per 7 days (3 unique users) in the histogram of Supplementary Figure 3 are due to different triggering mechanism modes activated on those users which collected more measurements over time. Importantly, the algorithm for BP estimation via photoplethysmographic signals was independent from the triggering mechanism modes. Consequently, different triggering mechanism modes did not impact on BP estimation throughout the study. This is supported by the BP values shown in Supplementary Figure 4 – no outliers BP values were found in the measurements. In other words, the additional readings collected with a different triggering mechanism introduced no measurement bias in the estimation of BP and did not impact in the comparison between the Aktiia monitor versus ABPM.

## Details for the exclusion of patients from the study

In total, 40 sessions and 12 patients were excluded from the present preliminary study. Supplementary Figure 5 illustrates the reasons for the exclusion of the 40 sessions:

- - At the start of the present preliminary study, 52 patients (out of the total 63 patients estimated to be recruited) had been recruited and were included in the analyses. These 52 patients had 90 sessions with ABPM and/or Aktiia data from the first and/or last week of CR program.
  - From the 90 sessions, 2 ABPM sessions were removed because they had fewer than 20 daytime measurements, remaining 88 sessions from 51 patients.
  - From the 88 sessions, 19 Aktiia sessions were removed because they had fewer than 20 daytime measurements, remaining 69 sessions from 49 patients.
  - From the 69 sessions, 19 Aktiia sessions were removed because they did not have a minimum of 7 days of data, remaining 50 sessions (35 from the first and 15 from the last week) from 40 patients. This cohort was used in the subsequent parts of the study.


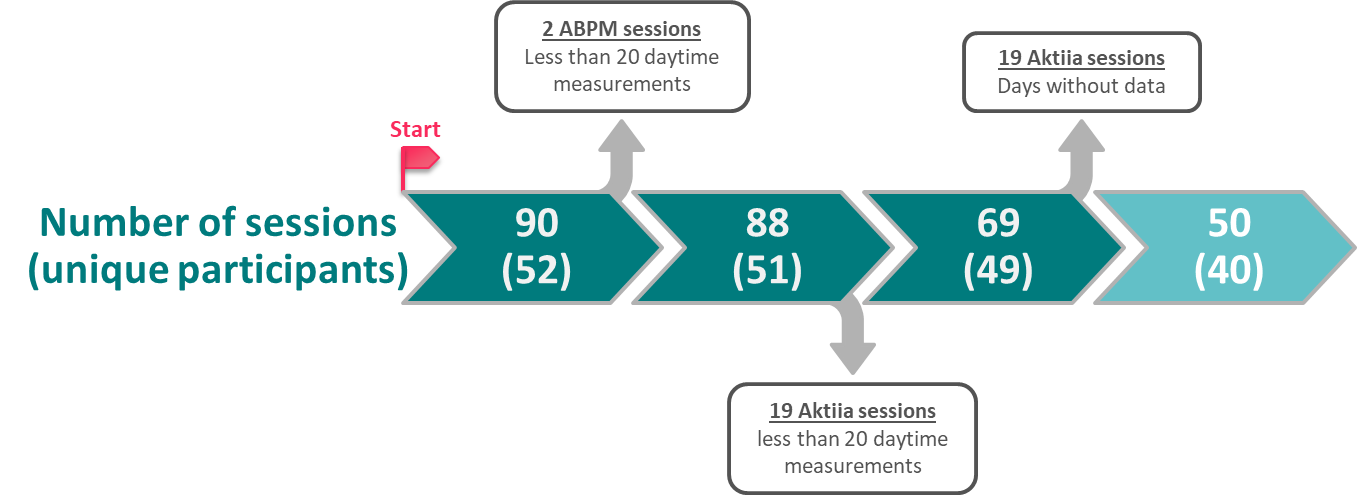


**Supplementary Figure 5**. Diagram detailing the flow of patients accepted to (and removed from) the study.

## Daytime BP measurements

Only daytime (9am-9pm) data was used in the analyzes. Supplementary Figure 6 shows a decrease on the number of measurements performed by the Aktiia monitor during this period of the day due to increased activity from the subjects. It is important to highlight, however, that the Aktiia monitor tries to collect data persistently. This means that, even if measurements are missed due to movement, the Aktiia monitor will keep trying to collect data. The Aktiia monitor has multiple embedded triggers that continually monitor the conditions to make sure measurements are performed under optimal circumstances. Nevertheless, if the participant does not rest over long periods of time or removes the bracelet – for instance, during practice of physical activities or removing bracelet for shower – the Aktiia monitor will be unable to perform measurements during those moments.

Despite this decreased number of measurements, the Aktiia monitor successfully performed 102.7 ± 60.8 daytime readings during the seven days of monitoring per subject, providing enough data for a comparison with ABPM.


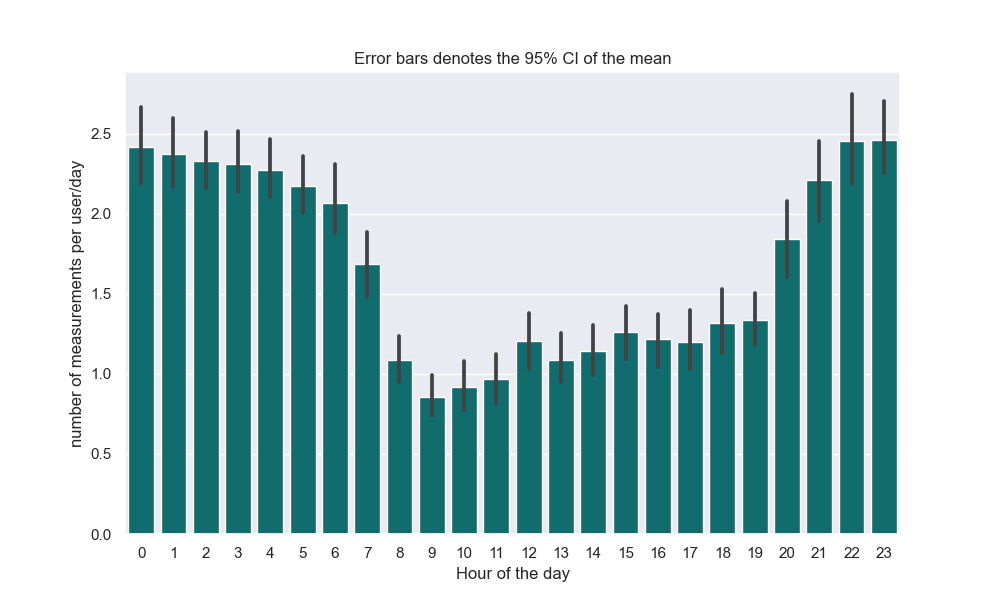


**Supplementary Figure 6.** The histogram of the number of data ($\mu\pm$ 95% CI) with time of day on the horizontal axis for the 50 sessions included in the analyzes.

In order to illustrate the fact the Aktiia monitor collects PPG data when the patient’s wrist is not moving and the patient is at rest, the differences in the 95^th^ upper percentile of SBP values measured by ABPM and Aktiia monitor considering the BP values collected within the same day have been calculated (Supplementary Figure 7A). The 95^th^ percentile was chosen to minimize the effect of potential outliers from the BP modalities. Similarly, Supplementary Figure 7B illustrates the differences in the 95^th^ percentile of SBP values measured by ABPM and Aktiia monitor considering the BP values collected within the same session. In other words, the 95^th^ percentile of SBP values measured by ABPM during one day was subtracted by the 95^th^ percentile of BP values measured by the Aktiia monitor during the equivalent week. Supplementary Figure 8 illustrates a similar analysis performed with DBP.

In all cases, ABPM demonstrated higher average BP values compared to the Aktiia monitor, with few instances where the Aktiia monitor have overestimated maximum BP values compared to ABPM. These results highlight the fact the Aktiia monitor collects PPG data when the patient’s wrist is not moving and the patient is at rest, while ABPM may monitor BP even when the wearer is actively moving, or just stopped from an effort.


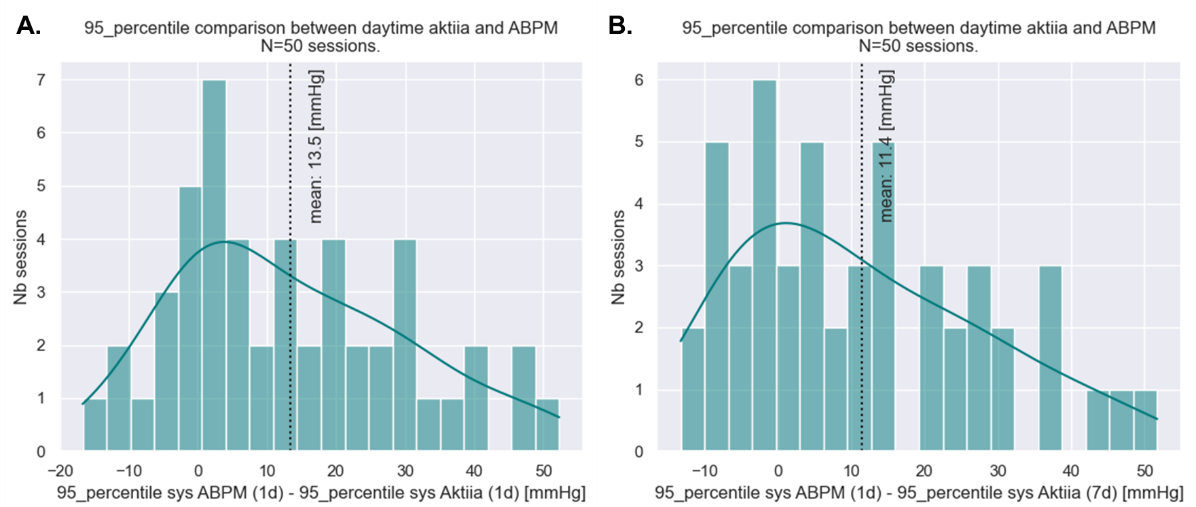


**Supplementary Figure 7.** Differences in the 95^th^ upper percentile of SBP values measured by ABPM and Aktiia monitor for BP values collected within the same day (**A**), and considering the BP values collected within the same session (**B**).


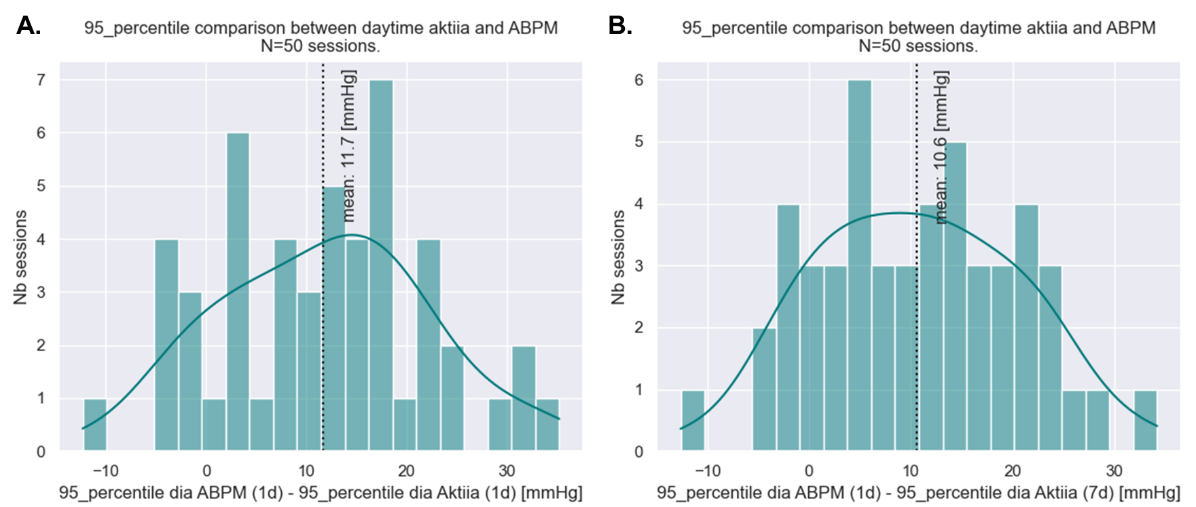


**Supplementary Figure 8.** Differences in the 95^th^ upper percentile of DBP values measured by ABPM and Aktiia monitor for BP values collected within the same day (**A**), and considering the BP values collected within the same session (**B**).

# References

1. O’Brien E, Parati G, Stergiou G. Ambulatory Blood Pressure Measurement: What Is the International Consensus? Hypertension. 2013;62:988-94.
